# ESR Bridges: imaging and treatment of ovarian cancer—a multidisciplinary view

**DOI:** 10.1007/s00330-024-11072-0

**Published:** 2024-10-18

**Authors:** Stacey Bryan, Andrea Rockall, Laura Tookman

**Affiliations:** 1 Department of Gynaecological Oncology, Imperial College NHS Trust, London, UK; 2 Department of Cancer and Surgery, Faculty of Medicine, Imperial College, London, UK; 3 Department of Radiology, Imperial College Healthcare NHS Trust, London, UK; 4 Department of Oncology, Imperial College Healthcare NHS Trust, London, UK

## Ovarian cancer—What is the clinical need to be addressed?

Multidisciplinary working is the cornerstone for optimal management of ovarian cancer with radiological assessment crucial throughout the patient pathway. Ovarian cancer is a term that encompasses pathologically distinct malignancies, all of which can have different patterns of spread and responses to treatment. The most common type of ovarian cancer arises from the epithelial cells of the fallopian tubes and ovaries (epithelial ovarian cancer, EOC); rarer subtypes and those arising from non-epithelial cells include ovarian germ cell tumours, ovarian stromal tumours and small cell carcinoma of the ovary. Management of all ovarian cancers is centred around combining surgical approaches with systemic anti-cancer treatment. Increased understanding of the biology and genomic make-up of ovarian cancer together with enhanced surgical techniques and more targeted treatments has led to paradigm shifts in the way that the disease is managed. Despite these advances, EOC continues to be the most lethal gynaecological malignancy responsible for over 200,000 deaths per year worldwide [1]. There is no effective population screening. Ovarian cancer is associated with non-specific symptoms and many women present late with advanced disease. There is an unmet need for earlier detection, novel and more targeted treatments and a better understanding of the development of treatment resistance.

### Challenges and opportunities—radiologist’s view

Adnexal masses are frequently diagnosed on pelvic ultrasound in symptomatic women, but also as an incidental finding on other imaging modalities such as CT, undertaken for a different purpose. Many adnexal lesions are benign and there are well-established diagnostic algorithms using ultrasound to categorise findings and stratify the risk of malignancy [2–4]. Approximately 20–25% of adnexal lesions remain indeterminate in the US and in these cases, MRI may help to improve the classification. The key is to ensure a high detection rate for malignancy whilst not over-treating benign lesions. One challenge is to avoid over-extensive surgery (cytoreductive cancer surgery) for benign lesions, which could render a pre-menopausal woman infertile or be unnecessary in a woman with high anaesthetic risk.

O-RADS MRI risk stratification is an evidence-based approach which uses a standardised MRI protocol, with functional sequences to categorise lesions into five risk groups and uses a standard lexicon for clarity of reporting [5]. The highest risk groups are score 4 (50% of which are borderline or invasive cancer) and score 5 (90% of which are borderline or invasive cancer). These patients should be managed in an ovarian cancer centre. Management of women with a score of 2 (almost certainly benign) and a score of 3 (95% likely benign) is decided on a clinical basis. Further research may improve the score, particularly in score 4 and borderline lesions.

In disseminated peritoneal disease, the key role of imaging is to provide a map of the extent of the disease, to plan the best treatment, as well as in assisting biopsy, response assessment and detection of relapse. The main challenge for radiologists is to detect small-volume disseminated disease, which can be difficult to detect on CT, including in sites that are considered more difficult to resect. The aim is avoidance of ‘open and close’ surgery when the disease is too extensive. MRI has been demonstrated to better delineate the extent of peritoneal disease [6] and may help to improve patient selection in future.

## Challenges and opportunities—other clinician’s view

The interplay between radiology, surgery, and systemic therapy has been instrumental in the diagnosis and treatment of ovarian cancer. Central to the management is surgical resection, with the aim to remove all visible disease. Unlike many other malignancies, surgical resection is often performed without a biopsy, as performing a biopsy on a solitary pelvic mass would risk upstaging the disease. The decision to proceed with initial surgery is determined by the radiological extent of the disease, with a particular focus on sites that are difficult to resect. Imaging criteria against surgical resection include disease involving small bowel mesentery, bowel carcinomatosis, involvement of stomach/duodenum and pancreas, coeliac trunk, and distant disease [7]. In these cases, a biopsy is required to determine the pathological subtype (and give further genomic information) to guide systemic therapy.

The addition of, or primary treatment with, chemotherapy and other anticancer treatments is determined not only by pathological subtype but also by stage (FIGO) of the disease. High-grade serous cancer is the most common subtype of EOC, it is characterised by complex genomic changes and approximately 50% have defects within the homologous recombination DNA repair pathway (including *BRCA1*/*2* mutations). This knowledge has brought germline genetic testing into the oncology clinic, and treatment with poly-ADP Ribose polymerase inhibitors is now part of care as maintenance treatment in advanced (and relapsed) ovarian cancer in those who have responded radiologically (and biochemically) to platinum chemotherapy, with the greatest benefit seen in those with *BRCA* mutations and homologous recombination deficiency [8].

## Future directions

The implementation of the O-RADS MRI score in the assessment of sonographically indeterminate adnexal masses means that fertility-sparing or even conservative approaches can be utilised in young patients with solitary masses. Adnexal lesions with a score of 2 are highly likely to be benign and can be managed accordingly. The added value of MRI is in the improvement of specificity, with the potential to reduce the number of women over-treated for a benign lesion whilst ensuring detection of malignancy as early as possible.

Similarly, whilst complete surgical clearance in advanced high-grade EOC is standard of care, fertility-sparing approaches can be carefully considered in young patients with low-grade disease or non-epithelial subtypes. Although similar chemotherapy regimens are often used in all subtypes of EOC, the rarer subtypes are underrepresented in clinical trials. New treatments have entered the clinical space; for example, targeting the mitogen-activated protein kinase pathway in low-grade-serous cancer [9]. Future directions of treatment for EOC include antibody-drug conjugates and immunotherapy [9].

Radiomics is a promising field within medical imaging which has the potential to act as a risk stratification tool in the management of ovarian cancer. Integrating predictive models of disease recurrence, prior to treatment into clinical practice, may increase individualised treatment and improve patient outcomes [10].

## Recommendations for clinical practice

- Implementing the use of O-RADS MRI risk stratification in the management of patients with adnexal masses to help reduce overtreatment.
- Be aware of and report on the sites of disease in ovarian cancer which may prevent surgical resection or may be difficult to resect which helps the multidisciplinary team to plan treatment.
- Recognition that advances in germline and tumour genomic testing allow greater options for patients with advanced and relapsed ovarian cancer and the importance of evidence of radiological response to chemotherapy in the selection of these treatments.

## Directions for future research

- Continued work to improve the specificity of the O-RADS score, particularly in the borderline group, which could guide selection for fertility-sparing approaches.
- Developing a combined tailored approach to management particularly in young patients with non-EOC or low-grade disease, including investigating novel treatment options.
- Use of radiomics and integration of predictive models of disease recurrence prior to treatment to improve outcomes.
